# Supervision of Surgical Trainees During Operative Procedures: Implementation of a Formal Preoperative Surgical Checklist

**DOI:** 10.7759/cureus.29466

**Published:** 2022-09-22

**Authors:** Ullas Jayaraju, Kueni Igbagiri, Christopher Brown, James Brock, Michael Cronin, Claire Carpenter, Rajesh Nair

**Affiliations:** 1 Trauma and Orthopaedics, Morriston Hospital, Swansea, GBR; 2 Trauma and Orthopaedics, Royal Surrey County Hospital, Guildford, GBR; 3 Trauma and Orthopaedics, Prince Charles Hospital, Merthyr Tydfil, GBR; 4 Trauma and Orthopaedics, University Hospital of Wales, Cardiff, GBR; 5 Trauma and Orthopaedics, Guy’s Hospital, London, GBR

**Keywords:** training and education, orthopedics and traumatology, iscp, interpersonal and communication skills, checklist development

## Abstract

Background

Trainers in surgery have an educational obligation to train trainees in performing operative procedures.

Objective

We hypothesized that poor concordance manifests as discrepancies between the trainee and the trainer, with an associated reduction in satisfaction with the training experience, perception of training quality, and completion of workplace-based assessments (WBAs). This study also aimed to validate the novel Supervised Training Operative Procedure (STOP) online tool.

Method

We developed an online proforma (STOP online tool) and conducted a prospective, single-blinded study of 53 orthopedic operative procedures with 53 trainees between January 19, 2019, and August 27, 2019.

Results

Forty-four (82%) trainees were listed as the primary surgeon. The overall mean trainee satisfaction (on a 0-10 Likert scale) was 8.25 (range: 3-10), and the mean trainer satisfaction was 8.28 (range: 4-10). A preoperative discussion between the trainee and the trainer occurred in 96.2% of the cases. Forty-eight (91%) trainers preoperatively established trainees’ objectives and 91% (n = 48) of the cases showed postoperative completion of objectives. Forty-four (83%) trainers anticipated workplace-based assessment (WBA) completion for trainees, and this translated into 41 (77%) completed WBAs. Overall, 47 (92.9%) trainees felt that the STOP tool would be useful as a surgical training checklist and in the completion of WBAs.

Conclusion

The STOP checklist is useful in understanding qualitative and quantitative measures of the overall trainee performance of an operative case. This holistic approach will enable us to establish a structured perioperative surgical training checklist, as trainee and trainer requirements are dependent on one another.

## Introduction

Surgery is a craft specialty, where the operative caseload is essential to produce a technically competent surgeon and thereby improve patient outcomes in independent practice. To be able to achieve these aims, the trainee must develop knowledge and practical skills through experience in the operating theater.

In the UK, the training standards for surgery are set by the Joint Committee on Surgical Training (JCST), which is composed of the specialty advisory committee (SAC), the Core Surgical Training Advisory Committee, the Training Interface Group, and the Intercollegiate Surgical Curriculum Programme (ISCP). The JCST is an advisory body to the four Royal Surgical Colleges serving the UK and Ireland concerning all aspects of surgical training [1-4].

The standards of surgical training in the UK have recently undergone changes, with the implementation of a new curriculum. This change reflects the response to the working patterns mandated by the European Working Time Directive (EWTD) and a shift in training to a competency-based approach with measurable objective outcomes [5].

A trainee’s development is assessed through a range of workplace-based assessments (WBAs), completed by their surgical trainer, and recorded on their designated online ISCP training portfolio [6,7]. Operative and technical skills are assessed with procedural-based assessments (PBAs) and direct observation of procedural skill (DOPS) assessments for several index procedures. The overall quality and success of a particular training event are frequently determined by the trainee-trainer relationship, which can be complex and varied. This can be influenced by case selection and complexity, operating list logistics, level of trainee or trainer experience, and other personal traits such as learning style compatibility and personality [8]. It is, therefore, necessary to establish realistic and achievable goals that can be delivered during the designated training interaction.

This study aimed to evaluate the level of concordance between a trainee and trainer’s expectations and agendas during operative learning episodes. The hypothesis was that poor concordance manifests as discrepancies between the preoperative goals of the trainee and the trainer, with an associated reduction in training experience satisfaction, perception of training quality, completion of WBAs, and completion of operative cases. The study also aimed to validate the novel Supervised Training Operative Procedure (STOP) online tool.

## Materials and methods

STOP online tool

A bespoke online assessment tool, created by the authors, was developed utilizing online forms. This is an online-based survey system that provides immediate/live responses to questions. Due to the large number of junior surgeons using electronic devices, we wanted to ensure that this proforma could be easily accessible and completed while in theater, on the ward, or in the clinic. This method was validated by the departments’ senior consultant orthopedic surgeons, who are trained educational supervisors. Completion of the National Health Service Research Ethics Committee (NHS REC) tool confirmed that ethical approval was not required for this project. Exploratory factor analysis was not used.

Completion of a WBA is done through the ISCP website. This is an extensive and comprehensive process that is not mobile-user friendly [9]. This is most often completed during a one-on-one between trainer and trainee when time is available. Due to the busy work schedule of both parties, these sit-down sessions can be difficult to create and subsequently result in a certain group of trainees not fully reflecting their skills, on ISCP, over an allocated period [10,11].

The STOP online tool aims to objectively analyze the interprofessional relationship between the trainee and the trainer. Compared with current checklist systems, we aimed to keep this online tool concise with the relevant objective data [6].

Participants

Inclusion criteria were any trauma and orthopedic procedures associated with a PBA or DOPS assessment on the ISCP portfolio and procedures where a National Training Number (NTN) trainee was being trained by an individual with a defined training role. Procedures were excluded if there were two operators of a similar training grade, non-NTN trainees, or cases undertaken in the private sector. Basic demographic data were collected for trainees and trainers (grade and specialty). Therefore, each case included one trainee and one trainer.

Data collection

Data were collected prospectively between January 19, 2019, and August 27, 2019. This was undertaken at a tertiary center and a district general hospital. Trainees and trainers were given access to the online data collection tool but were blinded to each other’s responses. Cases could be discussed en masse before starting a complete list or individually prior to each training case. Data collected from the online tool were transferred to a password-protected computer for subsequent analysis. No patient-identifiable data was included in the project.

Data analysis

A prospective, single-blinded, online-based proforma of 53 operative procedures carried out between January 19, 2019, and August 27, 2019, were reviewed. Prior to the operative procedure, a discussion was carried out between trainee and trainer (consultant and registrar, consultant and senior house officer, registrar, and senior house officer), which was verified with a checklist. This included a set of 13 preoperative questions (Tables 1, 2), followed by six postoperative questions (Table 3). This allowed for set criteria to be set between trainer and trainee. Targets were outlined including key steps the trainee is expected to complete, the percentage of the procedure the trainee is expected to complete, and whether a WBA would be completed.

**Table 1:** Preoperative trainee section This is the first section of the online Google Form, which is assigned to the trainee. The questions are as outlined. We ensured that the questions were able to yield objective results.

Trainer and trainee expectations of supervised operative procedures
Before commencement of case - trainee
Current surgical specialty and stage in training:
Name of operation:
Has there been a preoperative discussion regarding the case?
Yes
No
Has the trainee’s objectives for this case been established?
Yes
No
Name three key steps in the procedure you would like to perform:
Do you anticipate the completion of a WBA?

**Table 2:** Preoperative trainer section This section focuses on the trainer’s perspective of the trainee and establishing preoperative aims.

Trainer and trainee expectations of supervised operative procedures
Before commencement of case - trainer
Current surgical specialty and grade:
Has there been a preoperative discussion regarding the case?
Yes
No
Has the primary surgeon been established?
Trainee
Trainer
Has the trainee’s objectives for this case been established?
Yes
No
Do you anticipate the completion of the trainee’s WBAs?
Yes
No
What % of the procedure would you like the trainee to perform?

**Table 3:** Postoperative phase of the proforma This section identifies the trainee’s outcome and the trainer and trainee’s satisfaction.

Trainer and trainee expectations of supervised operative procedures
On completion of case - trainer and trainee
Who was the primary surgeon?
Trainee
Trainer
What % of the case did the trainee perform?
Has the trainee’s objectives for this case been completed?
Yes
No
Was there completion of the WBAs?
Yes
No
Trainee satisfaction of the case as a training/learning opportunity (1-10)
(Low) 1 2 3 4 5 6 7 8 9 10 (High)
Trainer satisfaction of the case as a training/teaching opportunity (1-10)
(Low) 1 2 3 4 5 6 7 8 9 10 (High)

## Results

A total of 53 procedures were recorded, comprising 53 individual training events between a trainee and a trainer. The most common procedures are displayed in Table 4. Most operations performed were secondary to hip fractures.

**Table 4:** List of procedures performed grouped by anatomical region (N = 53) IM: intramedullary nail; THR: total hip replacement; DHS: dynamic hip screw; TKR: total knee replacement; ORIF: open reduction internal fixation; POP: plaster of Paris

Anatomical regions	Procedures performed
Hip	Prophylactic IM femoral nail for metastatic disease (2), IM femoral nail (5), hip hemiarthroplasty (9), THR (3), DHS (7)
Knee	TKR (3), knee arthroscopy (2), tibial fracture ORIF (1)
Hand	Nail bed repair (3), Dupuytren’s contracture fasciotomy (1), trigger finger release (1), carpal tunnel decompression (4), distal radius closed reduction under sedation + POP application (1)
Elbow	Tension band wiring of olecranon fracture (1)
Shoulder	Closed reduction of anterior shoulder dislocation (3)
Foot and ankle	Ankle ORIF (6), ankle syndesmotic screw (1)

Trainee training grades included 42 core surgical trainees (CSTs) and 11 specialty registrars. These were divided into eight orthopedic core surgical trainee year 1 (CT1s), 33 core surgical year 2 (CT2s), four specialist trainee year 3 (ST3s), and seven specialist trainee year 5 (ST5s). Trainers were predominantly registrars (n = 25), followed by consultant orthopedic surgeons (n = 24). Trainer specialty registrars were separated into two specialist trainee year 8 (ST8s), four speciality trainee year 7 (ST7s), two ST5s, three speciality trainee year 4 (ST4s), and one ST3; 13 registrar grades were not specified.

Results of preoperative phase between trainee and trainer

A preoperative discussion between the trainee and the trainer occurred for 96.2% (n = 51) of the cases. Only 3.7% (n = 2) did not have a preoperative discussion. The primary surgeon was established in 83% (n = 44) of the cases; however, in 17% (n = 9) of the cases, the primary surgeon was not identified prior to starting the case. Of the trainers, 92% (n = 49) stated that objectives had been set for the trainees prior to the start of each case, and 85% (n = 45) anticipated completion of a WBA. With regard to performing an operation, preoperatively more than half the trainees (53% (n = 28)) wanted to perform the entirety of the operation. Six trainees anticipated 90%, six anticipated 75%, five expected 70%, and seven anticipated <50% completion of the operations.

Postoperative phase results

The trainee was the primary surgeon in 83% (n = 44) of the cases, with the trainer primary surgeon in 17% (n = 9). The trainee’s objectives were completed in 91% (n = 48) of the cases, and postoperatively, this also led to the completion of 41 (77%) WBAs. The proforma allowed comparison between the percentage of the operation the trainee aimed to complete versus the percentage of the operation that the trainee actually completed. Postoperatively, this amounted to 47% (n = 25) of the trainees performing the whole operation. Seven trainees performed 90% of their cases, four performed 80%, four completed 75%, three completed 70%, and 10 performed <50% of their respective cases. The mean for trainee satisfaction was 8.25 (range: 3-10), and that for trainer satisfaction was 8.28 (range: 4-10). The median value for trainee and trainer satisfaction was 9 and 8.5, respectively.

Overall, 88.7% (n = 47) of the trainees replied “Yes” to “Do you feel as though this system would help in the completion of a WBA and positive outcome?,” four said “No” (7.5%), and two (3.8%) did not respond. This mirrored 88.7% (n = 47) of cases answering “Yes” to “Do you think it would be useful to have as a surgical checklist?” Nonetheless, even with this positive feedback, there was a mixed response by trainees when asked, “Do you feel it would be more of a burden on training to have a surgical checklist?”; 51% (n = 27) answered “Yes,” 43% (n = 23) replied “No,” and 6% (n = 3) did not respond.

## Discussion

The world of surgery is changing [12,13]. The demands put on surgical trainees and the expected standards are ever-increasing. Simultaneously, training opportunities are shifting with requirements for surgical advancement set by the JCST, SACs, and NHS. The principal findings of this study are as follows: high levels of compliance for WBA completion (77%) and intraoperative trainee and trainer satisfaction (88.7%) and overall agreement that the STOP online tool would help in the completion of a WBA and positive overall performance outcome (88.7%). However, the trainees did acknowledge that, although this is a useful system, it would mean an increased burden on both trainees and trainers. Given that surgical training is shifting to a competency-based program rather than being defined by a fixed duration of training as the primary goal, we feel that identifying and targeting competencies could lead to a more efficient surgical training path. Therefore, the burden of performing these checks would be outweighed by the efficiency of progressing through the scheme faster.

In 83% of cases, the trainee was classified as the primary surgeon, with 83% of the operative cases being performed by the trainee. This is a reassuring finding, as it shows that the preoperative discussion or briefing (completed in 96.2% (n = 51) of the cases) can be a useful tool when conducted in the appropriate clinical setting. This allowed us to deduce that if the trainee and trainer work together, when each is aware of the goals, this leads to a focused, satisfactory, and productive training outcome.

Trainees of different training grades participated in the study, highlighting the diversity of individual opinions and training skills/abilities, which in turn influence the overall satisfaction of the cases. However, it must be said that the largest group of trainees was occupied by core surgical trainees (CSTs), with limited surgical experience, in comparison to registrars; nonetheless, this still led to the trainee being the primary surgeon in 83% of all cases. This is the desired outcome for junior surgeons to improve their operative skills.

The mean level of satisfaction for trainees was 8.4, compared with 8.3 for trainers. High satisfaction rates can lead to better training experiences and surgical outcomes. The fact that both scores are distinctly similar is a reliable indicator that the proforma was a useful training tool. The proforma provides a focused guide for establishing the required training outcomes for the trainee for each operation. If one can provide an environment that encompasses quality teaching, then the operative skills/experience will ensure high levels of compliance and the completion of WBAs.

Overall, both trainee and trainer had similar responses and expectations. Because of the constantly changing surgical environment, tools such as the STOP tool can be beneficial. Placements for core surgical trainees are usually divided into four- or six-month placements [14]. CSTs have to cover on-call/service provision shifts, leaving a relatively short period to be able to build an operational relationship with a trainer. The trainee may operate with several different trainers during their rotation [15,16]. Due to the continually changing environment, the STOP checklist provides an opportunity for the trainee to discuss a previous similar case with their current trainer, allowing for more structured goals.

Another advantage of this surgical training checklist is the ability to use the information on the proforma to aid in the completion of WBAs. The checklist will have a clear path preoperatively and postoperatively, highlighting the points the trainee wants to achieve from each operation, a more personalized approach to training.

Several studies in the literature focus on the attributes of trainers and the influence/impact they can have on improving trainees [17]. For instance, a well-established course held by the Royal College of Surgeons is “Training the Trainer” [18]. As we have mentioned, surgery is a craft specialty whereby tangible and intangible characteristics are required to make a well-rounded surgeon. At present, there does not appear to be another online tool, such as the STOP tool, assisting in surgical training and focusing on both trainee and trainer. The online proforma will consolidate knowledge for each case and improve trainee communication.

There are, however, limitations to this study. A voluntary checklist or questionnaire works best if participants have a good trainer-trainee relationship. Therefore, there is likely some unavoidable selection bias in our results. At the same time, it is unlikely that 83% of the procedures in all orthopedic departments are being done by the trainee rather than the trainer, highlighting the limitations of a voluntary checklist. The cohort of patients in this study is a good representative of orthopedic surgery; however, a larger dataset would yield more reliable results. Moreover, the recruitment of patients from other surgical specialties would allow us to assess interprofessional variations. At present, the STOP online proforma does not consider previous operative achievements by the trainee. Each time the proforma is accessed, it is considered a new event. Therefore, this may not fully represent the trainees’ experience, especially when they encounter a new trainer.

Although this study has some limitations, a preoperative and postoperative surgical training checklist is a useful tool to develop the quality of a surgical training event. Further analysis in other surgical departments with a larger cohort would aid in determining the benefit of this tool in other surgical specialties.

## Conclusions

Our aim was to understand the perspectives of both the trainee and trainer during each operative case and formalize the results. We found that the STOP checklist is a useful tool to understand the overall quantitative experience of a trainee’s performance in an operative case and in the completion of a WBA. This holistic approach will enable us to establish a structured perioperative surgical training checklist, as trainee and trainer requirements are dependent on one another.
